# Passive nuclear transport deviates from Fickian behavior in prostate and breast cell types

**DOI:** 10.1080/19491034.2026.2620223

**Published:** 2026-01-31

**Authors:** Nicholas R. Scott, Alexander J. Lin, Brian Belardi, Sapun H. Parekh

**Affiliations:** a Department of Biomedical Engineering, University of Texas at Austin, Austin, TX, USA; b McKetta Department of Chemical Engineering, University of Texas at Austin, Austin, TX, USA

**Keywords:** Cancer, nuclear trafficking, size-dependent transport, nuclear transport, membrane diffusion, transport in cancer

## Abstract

Nuclear trafficking is essential for cellular function and biomedical applications such as nucleus-targeted drug delivery; however, how passive nuclear transport varies across cell types and phenotypic states remains poorly understood. Here, we investigate passive nuclear transport of fluorescent molecular cargoes spanning 500–20,000 Da across multiple cell lines. We observe cell-line-specific nuclear restrictions and find that passive nuclear uptake does not exhibit a monotonic dependence on molecular weight, suggesting non-Fickian transport behavior. Furthermore, transforming a healthy breast cell model into an invasive-like phenotype via TGF-Beta treatment significantly altered passive nuclear transport characteristics, closely resembling those of a well-established invasive breast cancer cell line. These phenotype-dependent changes in nuclear permeability provide new insight into fundamental biophysical alterations associated with cancerous cellular transformation.

## Graphical abstract

**Figure:**
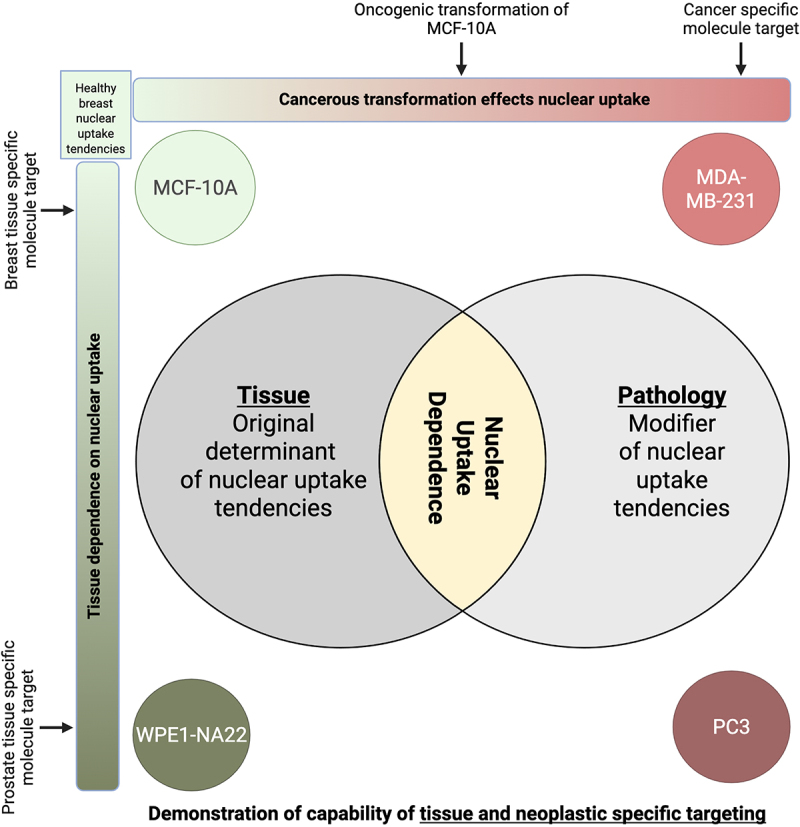

## Introduction

Passive nuclear transport is integral for small molecules and proteins that maintain normal cell behaviors. By traveling through nuclear pore complexes (NPCs) to traverse the double bilayer nuclear envelope, such cargo undergoes nucleo-cytoplasmic transport. While very small, minimally polar, or nonpolar molecules may traverse the nuclear envelope directly, without the use of the NPC [1], most transport occurs via the NPCs [2]. Passive diffusive transport into or out of the nucleus has been assumed to essentially obey Fickian diffusion up until a soft cutoff, where larger cargo (that above the cutoff) passive transport across the NPC becomes inefficient [2,3]. Instead, larger molecules (greater than ∼ 40 kDa), typically require nuclear transport receptors, such as importin-β or other karyopherins, to attach to the cargo’s nuclear localization sequence (NLS) to facilitate *active* transport into the nucleus [4]. However, certain large molecules possess specific molecular properties that allow them to passively enter the nucleus through the NPC without an NLS [5,6], in contrast to the majority of larger molecules, which exhibit minimal passive transport–highlighting the complexity of passive nuclear entry.

Further complicating the picture of passive transport is that different cells may exhibit different nuclear transport properties for reasons such as cell cycle or cell state. For example, earlier work showed that number of NPCs is both cell-line specific and cell-cycle dependent [7–11]. NPC constituents are dependent on cell type and differentiation, and NPC component mutations are involved in different diseases [12]. As an example, mutations in nucleoporins in yeast can make diffusive transport non-Fickian [3]. Therefore, investigating how cargo size, tissue origin, and pathology state affect passive diffusion into the nucleus is clearly relevant. However, many experiments often use only one cell line, which has overlooked this heterogeneity. Here, we explore size-dependent effects on passive transport across various cell types by investigating the passive diffusion of cargoes with different molecular weights into the nucleus. This introductory study includes four cell lines derived from two different epithelial tissues, containing both normal and cancerous states for each tissue, as a means to demonstrate cell-specificity of passive nuclear transport and is not intended to be an exhaustive library.

Passive nuclear transport’s physiological relevance is multifactorial. Passive diffusion through the NPC allows for the bidirectional movement of small molecules, proteins, and signaling molecules below a defined size threshold, enabling rapid responses to extracellular and intracellular cues [3]. Therefore, passive transport plays a critical role in processes such as transcriptional regulation [13,14], stress response, and development, with changes in NPC composition or permeability observed across cell states and many disease contexts [12,15]. Protein mislocalization or relocation may change the protein’s function within the cell, even leading to aggregation or other behaviors that precipitate a pathology [16]. Pharmacologically, nuclear permeability is a restriction within the cell that drug delivery must overcome if the drug has a nuclear target (such as doxorubicin). Juhlen et al. [15] note that nucleoporin (the protein subunits that constitute the multi-megadalton nuclear pore complex) mutations lead to tissue-specific diseases. Additionally, nucleoporins aid in both differentiation and metabolic activity [12,17]. Studies have also shown that epithelial-to-mesenchymal transition (EMT), a key event in tumor progression, is associated with an altered trafficking of large nuclear-localized proteins requiring facilitated transport [16]. Thus, the biological relevance of change in passive uptake is vast.

Despite these previous findings, relatively few studies have directly compared passive nuclear permeability across multiple cell types or transformation states. Here, we investigate passive nuclear transport of small-molecule cargoes with various molecular weights across the NPC in different cell lines and in response to EMT. While our study only uses four cell lines, we hope our efforts and results stimulate future work to examine the cell-type specificity of nuclear transport. Building on our previous work studying passive nuclear transport of chemotherapy in aggressive prostate and breast cancer cells, we employ a similar methodology to isolate nuclear transport in the current study to measure molecular cargo’s nuclear uptake, where the molecular weights (MW) range between ∼ 700 Da (as a non-reactive fluorphore) to 10 kDa (a fluorescently tagged dextran). To bypass the plasma membrane for all cells, we perforate the cell membrane with a pulse of digitonin, a detergent that has been used extensively for plasma membrane permeabilization and has been shown to maintain nuclear intactness [6,18–20]. Moreover, we verify the robustness of the results obtained through the use of streptolysin O (SLO), an entirely orthogonal cell membrane perforation method that also preferentially perforates the plasma membrane over the nuclear membrane [5,21,22]. Based on the data that follows, we find that traversing the NPC passively appears to be non-Fickian, as increasing molecular weight cargoes do not follow a monotonic reduction in transport as would be expected for pure diffusion. Additionally, breast and prostate tissues have distinctly different rates and amounts of uptake at a given end time for the various MW cargoes, and surprisingly, whether the cell is of cancerous or normal origin affects each tissue in differing manners. Our findings underscore the relevance of cell line–specific evaluation of nuclear trafficking, as assuming uniform behavior across different cell types may lead to inaccurate conclusions.

## Materials and methods

### Digitonin and DOX stock solution preparation

Digitonin (Millipore catalog # 300,410) was prepared in cell culture grade H2O (autoclaved UltraPure Type 1) and sterile filtered at a concentration of 10 mg/ml as solubility in water is around 50 mg/ml and was aliquoted to be stored at −20 degrees Celsius to avoid freeze thaws. Working concentrations of digitonin was 25–40 μ g/ml for any cell line (diluted in PBS no Calcium–Fisher Scientific catalog # MT21031CV–to restrict membrane repair), and the proper concentration was determined by having at least 60% permeabilization with at least 30% of the cells having a distinct and well defined nuclear staining. To stain the nuclei, RedDot2 (Biotium catalog # 40,061) with a dilution ratio of 1:200 should be prepared prior to experimentation within the digitonin solution. Additionally, DOX (TCI chemicals catalog # D4193) was dissolved in culture grade $H_{2}O$ at a concentration of 10 μM to be able to see rapid fluorescence intensity increase in the short permeabilization window of 20 minutes, as a similar protocol to our other work [23]. DOX should also be aliquoted at a higher concentration than working and stored in the freezer for single use purposes as well.

Digitonin permeabilization:

1. Normal media is replaced with $CO_{2}$ independent L-15 media for the duration the cells are out and not actively being permeabilized. The cell dish is placed in a heated stage pre-heated to 37 degrees Celsius
2. Wash cells 2x with PBS with no calcium
3. Incubate with digitonin-RedDot2-PBS no Ca cocktail incubate for 8 minutes
4. Wash 2x with PBS with no calcium
5. Ensure Zero Drift Compensator on the microscope is active
6. Start imaging process (if it is either free dye Alexa 488, fluorescent dextran, due to rapid diffusion rates)
7. Add fluorescent probe (either free dye Alexa 488, fluorescent dextran, or DOX) solution
8. Replace the two covers to ensure even heating
9. Start imaging process (if it is DOX, due to slow diffusion rates)

*Note*: DOX concentration was required to get sufficient fluorescence in a 20 minute timespan for permeabilized cells. Unpermeabilized cells did not have detectable fluorescence at our imaging parameters over the experimental time.

### Cell culture

MDA-MB-231 (female breast cancer line, ATCC catalog # HTB-26, $\mathrm{RRID}:\mathrm{CVC}L_{0}062$) was grown in complete media of DMEM and 4.5 g/L glucose (Corning catalog # 10–013-CV) with 10% FBS and no antibiotics. PC3 (male prostate cancer line, ATCC catalog # CRL-1435, $\mathrm{RRID}:\mathrm{CVC}L_{0}035$) was grown in complete media of RPMI 1640 (Gibco catalog # 11,875–093) with 10% FBS and no antibiotics. MCF-10A (female breast fibrocystic line, ATCC catalog # CRL-10317, $\mathrm{RRID}:\mathrm{CVC}L_{0}598$) was grown in DMEM/F12 (thermofisher catalog # 11,320,033) with 5% horse serum, no antibiotics, 20 nanogram/ml of epidermal growth factor, 0.5 mg/ml hydrocortisone, 100 nanogram/ml cholera toxin, and 10 microgram/ml insulin. WPE1-NA22 (normal prostate line, ATCC catalog # CRL-2849, $\mathrm{RRID}:\mathrm{CVC}L_{3}810$) was grown in Keratinocyte Serum Free Medium kit (K-SFM), (ATCC Kit catalog # 17,005–042).

Cell lines would be passaged and plated onto an 8-well ibidi slide (Ibidi Catalog # 80,806), where they would grow until they reach 80% confluence or greater, and then would be used in an uptake experiment. Cell lines were not explicitly verified using STR profiling and were not checked for mycoplasma contamination, however the passage numbers of any cell line purchased directly through ATCC would not exceed 10 passages for any experiment. Only the central 4 wells were utilized for dynamic uptake studies to ensure near-identical well temperatures by the heated stage and ensure that DOX uptake was not modified by heated stage based temperature fluctuations. All wells would be analyzed in one day to prevent infection risk. L-15 $CO_{2}$ independent and phenol-red free media (Gibco catalog # 21,083–027) was used for a short duration (typically less than 1 hour) during the fluorophore probe measurement process for any cell line to prevent pH imbalance before permeabilization. This was due to the open air gas exchange during the permeabilization process not being suitable for $CO_{2}$ dependent media in wells not being tested at that moment. Cells were not randomized and the investigators were not blinded to the cell line during experiments.

TGF-β 1 (R&D Systems catalog # 246-LP) was dissolved in sterile water, 7.5% BSA (Thermo # J10857-18), and HCl for pH as per the manufacturer’s guidelines, then subsequently aliquotted and stored in −80 degrees Celsius for single use. TGF-β 1 was incubated with MCF-10A cells for five days to induce transformation based on previous work [24,25] at a working concentration of 10 nanograms/ml in complete media. Refreshing media maintained the same concentration of TGF-β 1.

*0.1 GUV analysis*

GUVs were produced by electroformation with Nanion Vesicle Prep Pro. Briefly, lipids were dried on an ITO glass slide to produce GUVs with 68.9 % DOPC (Avanti Polar Lipids catalog # 850,375), 30 % cholesterol (VWR catalog # 0433), 0.1 % Liss-Rhodamine PE (Avanti Polar Lipids catalog # A81150) and 1 % (Avanti Polar Lipids catalog # 880,129). GUVs were electroformed with a 10 minute ramp up step, followed by a 10 V, 10 Hz condition for 2.5 hours, and finally a 10 minute ramp down step. After hydration with HEPES saline (25 mM HEPES pH 7.5 and 150 mM NaCl) (Sigma-Aldrich catalog # H3375), GUVs were incubated on a glass slide passivated with biotinylated BSA (Thermofisher catalog # 29,130) and 10 μ M Streptolysin O (Sigma catalog # SAE0089-100KU) at 37 C for 15 minutes. Wells were then washed five times by adding and removing 100 μ L of HEPES saline, in the final wash, 120 μ L was removed before imaging. To measure dye uptake into vesicles, 20 μ L of dye was added to each well to reach a final concentration of 10 μ M.

Images of the dye uptake were taken every 25 ms using a Dragonfly Andor spinning disk fluorescence microscopy system. The dyes were excited by the 488 laser line at 20 % power. Uptake was monitored by averaging the dye intensity within the GUV and dividing it by the intensity outside the GUV over time (*N* = 3 GUVs). Data was normalized to the maximum intensity per frame.

### Fixation and antibodies

For all antibody staining seen in Figure 5: 4% paraformaldehyde (Sigma # 158,127) was used to fix the cells, which were then subsequently incubated for 15 minutes at room temperature. Cells were washed thrice in PBS, then incubated for 10 minutes at room temperature on a shaker with 0.1% triton-x 100 (Fisher Scientific Catalog # 50–112-1262) and 0.5% BSA in PBS. Lamin A/C antibodies conjugated with Alexa-488 were purchased via Abcam (catalog # ab205769, $\mathrm{RRID}:AB_{3}105823$) and used at a concentration of 1:400 for ICC in conjunction with 0.1% triton-x 100 and 3% BSA for blocking for two days at 4 degrees Celsius. Nucleoporin-153 (Nup-153) antibodies conjugated to Alexa-647 were purchased via Abcam (catalog # ab204457-1001) at a concentration of 1:100 for ICC was simultaneously incubated with the lamin A/C antibodies. Two washes were followed to remove non-specifically bound antibodies with PBS, 0.1% triton, and 0.5% BSA before imaging.

Cytoskeletal staining was done simultaneously as well using the same method as above, but not in conjunction with the lamin A/C and Nup-153. Microtubules were stained with alpha tubulin antibody Alexa-488 (Thermo Catalog # 53–4502-82, $\mathrm{RRID}:AB_{1}210525$) using a 1:100 dilution ratio, and actin was labeled by phalloidin-594 (AAT Bioquest catalog # 23,122) using a 1:400 dilution ratio as per manufacture guidelines.

Imaging and staining conditions were done simultaneously for TGF-β 1 and control conditions of MCF-10A No counterstain was used to prevent FRETing.

### Fluorescent probes and permeabilization methods

Non-reactive, carboxylic-acid Alexa 488 free dye (Thermofisher catalog # A33077) was used throughout the paper as the smallest fluorescent probe, and was used as an inert molecular model analogue for small drugs. The Alexa-488 fluorescent dextran probes: 3kDa (Thermofisher catalog # D34682), and 10kDa (Thermofisher catalog # D22910) were used as larger molecular weight inert molecules. All inert fluorescent probes were used at a 10 μM concentration of the molecule.

The same digitonin protocol was used in (Fig. S2), but a similar permeabilization protocol but replacing digitonin with streptolysin O (Millipore catalog # S5265) with a 15-minute incubation time, similar to researchers Walev et al. [22], and Teng et al. [21].

### Confocal microscopy of nuclear uptake and antibody staining

An Olympus Fluoview FV3000 ($\mathrm{RRID}:\mathrm{SC}R_{0}17015$) was used for all imaging purposes and a Pecon Temp-Controller 2000–2 was used to ensure an adequate body temperature was maintained throughout the entirety of the experiment. For all live cell experiments, microscope settings were maintained across cell lines and conditions for any given molecule type, but may differ across molecule type (such as timespan of an experiment). DOX solubilized in cell-grade water was excited using the 488 laser line at 2% power and 600 HV and captured using a filter that allows 570–620 nm wavelength light to pass through to prevent significant photobleaching over the span of 20 min, where an image was taken every 6.43 seconds. Simultaneous imaging of DOX and RD2 was possible due to the excitation and emission differences, where RD2 was excited at 640 and its fluorescence was captured at 657–757 nm with a laser power of required for each individual cell line to get a good ROI of multiple nuclei without getting background. Seeing as this dye was only for nuclei mask image segmentation purposes, varying these laser parameters was not a concern. Similarly, the Alexa studies used 1.5% laser power and 400 HV, and captured using a filter that allows 500–540 nm wavelength light where the timespan of the experiment was 45 seconds, images were taken every 0.56 seconds. For the Dextran studies, 1.5% laser power and 400 HV, and captured using a filter that allows 500–540 nm wavelength light where the timespan of the experiment was two minutes and 10.26 seconds, images were taken every 1.08 seconds regardless of dextran weight.

All images were taken using a 20x air UPlanSapo 0.75 NA with varying a resolution size due to acquisition speed needs. The same objective was used for all Alexa, RD2, and DOX time lapse studies. The Olympus ZDC autofocus unit was used for all timelapse imaging studies to ensure the same focal plane was studied.

For fluorescent antibody studies using anti-lamin A/C, a 488 laser line to excite at a power of 1.5% and an HV of 570, collecting a z-stack throughout the entire thickness of the nuclei in the field of view to ensure nuclei at different heights were accounted for without bias. Anti-Nup-153 used a laser line of 640 at a power of 4.0% and an HV of 725. For cytoskeletal staining, actin’s 594 phalloidin used the 561 laser line at 1.5 laser power percent, and an HV of 460, whereas the anti-microtubule used the 488 laser line at 2% power and an HV of 425. No nucleus counter-stain was used to ensure FRETing did not occur to any extent, as DRAQ5 made fluorescence slightly weaker. These images were taken using a 40x air UPlanSapo 0.95 NA and an image size of 1024 × 1024 for nuclear antibodies, and 640 × 640 for cytoskeletal structure.

### Image processing

Post processing was done through ImageJ (version 2.16.0 $\mathrm{RRID}:\mathrm{SC}R_{0}03070$) for DOX or CellProfiler (version 4.2.8, $\mathrm{RRID}:\mathrm{SC}R_{0}07358$) for Alexa and dextrans. For FIJI, images were background subtracted, then the image was blurred to create an ideal gray image for auto-thresholding. Auto-thresholding used the Otsu method to identify nuclear objects, and two populations were captured using the auto-thresholding method a second time whilst ignoring the extra bright nuclei that were captured during the first auto-thresholding object detection. A macro script was built to allow for the same process to be used across cell lines, and treatments, reducing bias, while obtaining a very high capture rate of both of the different nuclei populations, this was previously lightly discussed in our other paper [23]. Creating three populations was explored, but background and cytosol was regularly captured in the third, indicating that there was not a significant population of nuclei that were not captured in steps 1 and 2.

For cell profiler, a similar setup was completed, but only captured the brightest group of nuclei, auto-thresholded and size constrained to reduce permeabilization bias of these impermeable molecules. Area, circularity, and fluorescence intensities were obtained, to create correlation graphs seen in Figure 5.

## Results and discussion

We previously observed distinct doxorubucin (DOX) nuclear uptake for purified nuclei from different cells [23], leading us to wonder about whether different cell lines harbored unique preferences for passive nuclear uptake of other small- or medium-sized cargoes. Here, we study if multiple cell lines from different tissues and pathologies have distinct passive nuclear uptake rates for cargoes at or below 10 kDa. Table 1 is a summary of all cell lines studied comparing their tissue origin, disease state, and growth phenotype.

**Table 1.** Cell lines analyzed in this passive transport dynamics study. Invasivity and tumorigenicity is described by and obtained from the ATCC website.

Cell Lines				
	MCF-10A	MDA-MB-231	PC3	WPE1NA-22
Tissue	Breast	Breast	Prostate	Prostate
Cell Model	Healthy	Cancerous (Grade III adenocarcinoma)	Cancerous (Grade IV adenocarcinoma)	Healthy
Morphology	Cobblestone	Spindle	Both	Both
Invasivity	N/A	High	High	Very low
Tumorigenic	No	Yes	Yes	Yes

To test the hypothesis that nuclear uptake rates may differ among cell lines, we utilized our previously established pulsed digitonin protocol to permeabilize the cells and introduce cell-impermeant fluorescent probes of varying molecular weights. During the 8-minute permeabilization process, we expect the loss of most small, cytosol-soluble molecules [26], while larger molecular assemblies and cytoskeletal structures remain. The fluorescent probes are introduced immediately following permeabilization step and are present for a short duration (∼ 2 minutes). Given this timescale, any observed differences in nuclear uptake are unlikely to result from interactions between the probes and residual cytosolic components that may vary between cell types.

**Figure 1.**
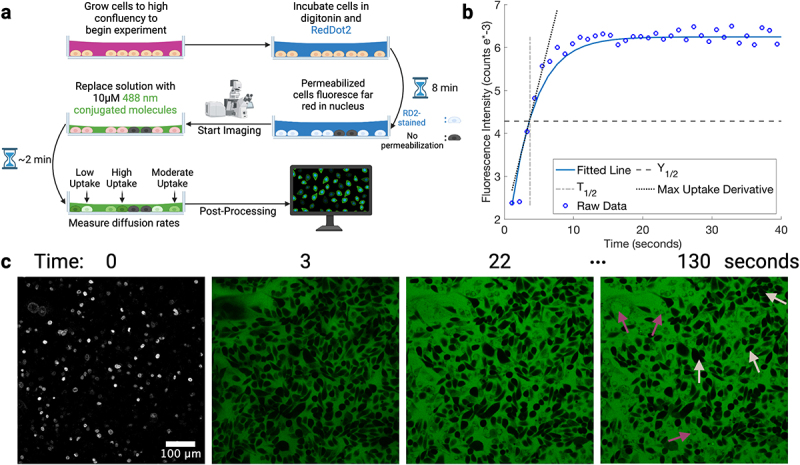
Experimental overview to measure fluorescent Alexa-488 labeled molecules’ nuclear uptake in cells. (a) Graphical representation of experimental setup. (b) Example of an individual nucleus’ intensity increase in the cargo channel. Raw data are circular points while the blue line is a polynomial best fit used to extract the, while the maximum rate of nuclear uptake was found by obtaining the derivative of the best fit line along every point of the curve. The dashed black line is the or half of the total uptake of the molecule for that given nucleus, whereas the dotted gray vertical line is the , or the time at which half of the uptake has occurred; both are used to obtain population statistics displayed in later figures. The y-axis on this graph is based on the post-processing software CellProfiler that use a 0–1 re-scaled fluorescent counts based on the maximum possible number associated with the bit count (for a 16 bit image it scales of the maximum of 65,535). The experimental time extends past where the graph cuts off. Here, we focus on early time points to graphically emphasize the quantities used for statistical comparison. (c) Exemplary images of the expierment at different times. Time 0 shows the RedDot2 channel that highlights (bright) nuclei that will be used as ROIs. This is before and cargo has been added. The three green colored images are of the Alexa-488 conjugated 10 kDa dextran channel at the indicated times. Passive nuclear uptake is seen in permeabilized cells at longer times (marked by dark pink arrows). Dark cells in the later time points (marked by light pink arrows) correspond to non-permeabilized cells. Figure compiled in BioRender.

We test if: (1) cell lines show distinct size-dependent nuclear transport and (2) nuclear transport in all cell lines generally follow Fickian diffusion for different MW cargoes, i.e. if larger cargoes show slower passive nuclear transport. Figure 1(a,b) show the experimental setup and population metrics extracted from our experiments. The nuclear fluorescence intensity (F.I.) of each cargo was normalized by the F.I. from a peri-nuclear ring established after nuclear segmentation. The peri-nuclear region was created by expanding the nucleus mask just beyond the nuclear boundaries but still within the cell boundaries, which allowed us to account for differences in photobleaching, digitonin permeabilization, and cargo availability on a cell-by-cell basis.

The Equation (1) below is pertinent to understanding our study, where F.I. stands for fluorescence intensity. While we optimized our digitonin protocol per cell, with this equation, extent of digitonin permeabilization should be canceled out *for each individual cell*. This cancellation occurs because the amount of cargo that surrounds the nucleus (the $F.I._{\mathrm{peri}-\mathrm{nuclear}}$ for a given cell would depend on the number of digitonin-induced pores, and the nuclear intensity would depend on both the number of digitonin pores and the peri-nuclear intensity:

(1) $$\frac{F.I._{\mathrm{nuclear}}}{F.I._{\mathrm{peri}-\mathrm{nuclear}}}$$

Successful permeabilization is marked by nuclear staining (later to be used as a nuclear mask) by an impermeant nuclear DNA staining dye–RedDot2 (RD2), see Figure 1(c) time point 0. As seen in the representative data in Figure 1(c), even the largest cargo, a 10 kDa dextran, enters the nucleus rapidly after introduction for permeabilized cells. Examples of non-permeabilized cells are the completely black cells in the field of view, which look identical to cells incubated with a fluorescent molecule without a prior digitonin pulse.

We use three metrics: nuclear $T_{1/2}$, normalized $Y_{1/2}$ ($Y_{{1/2}_{\mathrm{nuclear}}}/Y_{{1/2}_{\mathrm{peri}-\mathrm{nuclear}}}$), and the maximum derivative of the fitted line of the raw data to determine the maximum nuclear uptake rate, all depicted in Figure 1(b). These are used to capture the complexity of molecular uptake via nuclear fluorescence. Each metric provides unique insights: $T_{1/2}$ alone cannot distinguish between rapid uptake and lack of uptake; the uptake rate does not indicate total nuclear cargo; normalized $Y_{1/2}$ accounts for overall cargo accumulation, and may depend on how well the dye packs inside the nucleus. Using all provides a more comprehensive assessment of nuclear uptake dynamics.

### Ratiometric comparison of MW cargoes shows non-Fickian behavior

**Figure 2.**
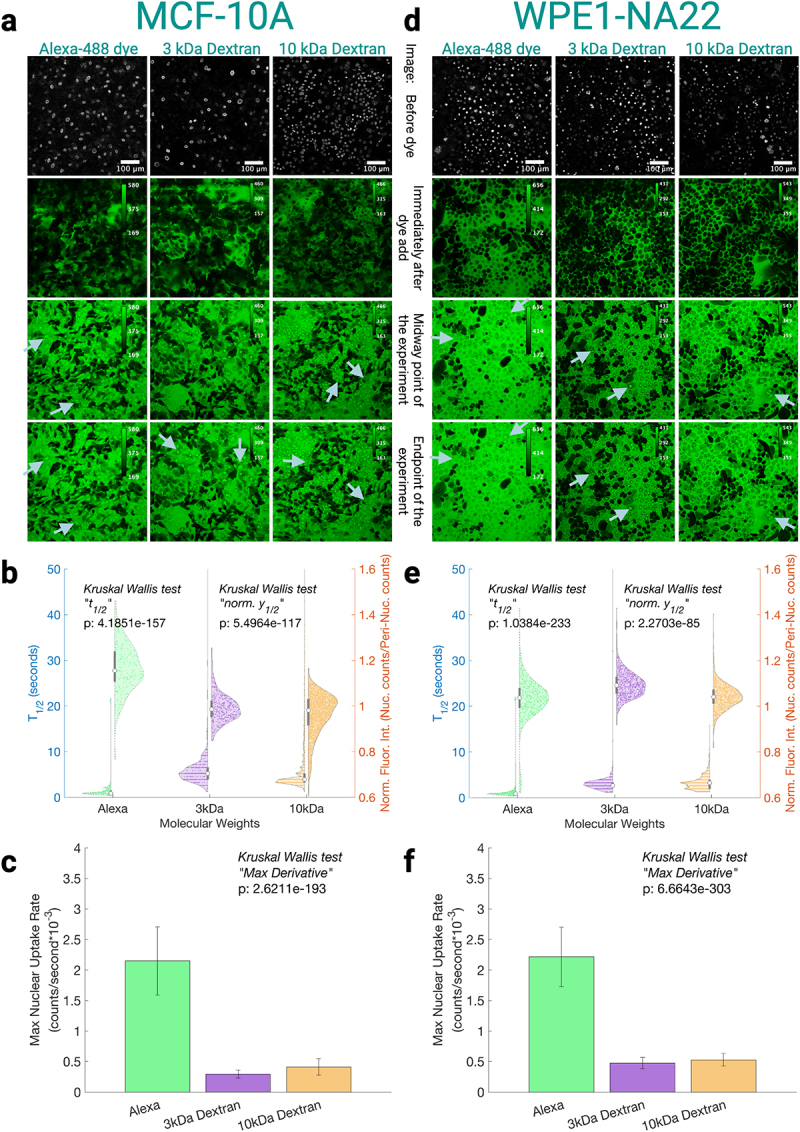
Free dye Alexa-488, 3, and 10 kDa fluorescent dextrans are used as probes to measure passive uptake of various MW into the nucleus in healthy cell models for respective tissue types. (a, d) Representative examples of fields of view for each cell line and each Alexa-488 conjugated dextran and the free dye Alexa-488. The first image for every molecular weight shows the nuclei of successfully permeabilized cells that will be used as ROIs for quantification. The images are ordered chronologically, where the second image is right after dye introduction, the third is the middle time frame, and the last is the final time frame within the experiment. For Alexa-488 experiments, the total time-span of the experiment is approximately 45 seconds, but for the dextran experiments the time-span of the experiment is approximately 130 seconds. Each MW is auto-contrasted in the fields of view to show differences in field of views while having an adjusted LUT for better ease of viewing, with the calibration bar in the top right of each pane. Arrows depict nuclei that have unique packing densities which correspond to their normalized to depict cellular heterogeneity, and how heterogeneity may differ across MW for a given cell line. (b, e) the population metrics were found as referenced in Figure 1b. The left hand half-violins of each MW correspond to while the right hand half violins of each MW correspond to the normalized fluorescence intensity ($Y_{1/2_{\mathrm{nuclear}}}/Y_{1/2_{\mathrm{peri}-\mathrm{nuclear}}}$). (c, f) Median behavior of the max derivative of the fitted line of each nuclei for all nuclei of a given cell line and MW, where the error bars are standard deviation; note the counts are CellProfiler re-scaled fluorescent counts rather than raw counts from the microscope. Number of cells and experiments are grouped by cell line; MCF-10A: alexa-488 *N* = 4 and *n* = 243 cells, 3 kDa *N* = 4 and *n* = 432 cells, 10 kDa *N* = 11 and *n* = 2,826 cells; WPE1NA22: alexa-488 *N* = 4 and *n* = 594 cells, 3 kDa *N* = 3 and *n* = 634 cells, 10 kDa *N* = 4 and *n* = 1105 cells. Figure compiled in biorender.

**Figure 3.**
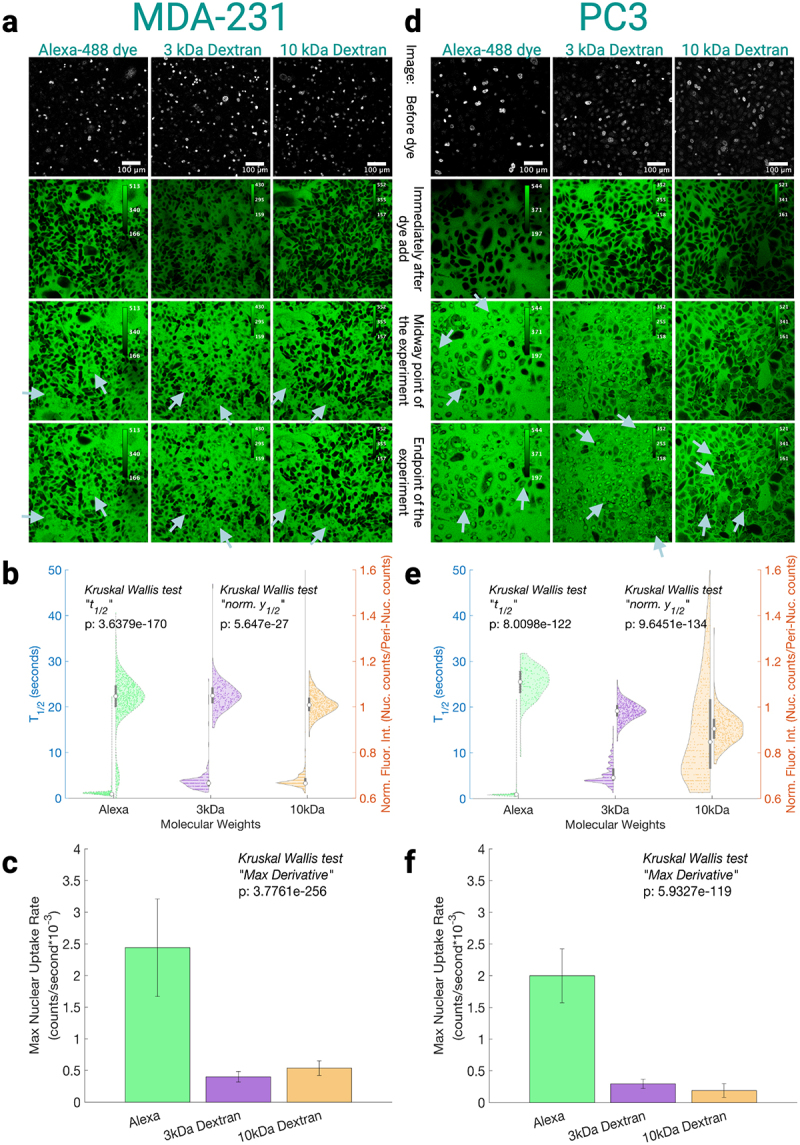
Free Alexa-488 dye, 3, and 10 kDa fluorescent dextrans are used as probes to measure passive uptake of various MW into the nucleus in cancerous cell models for respective tissue types. (a, d) Representative examples of fields of view for each cell line and each Alexa-488 conjugated dextran and the free dye Alexa-488. The first image for every molecular weight shows the nuclei of successfully permeabilized cells that will be used as ROIs for quantification. The images are ordered chronologically, where the second image is right after dye introduction, the third is the middle time frame, and the last is the final time frame within the experiment. For Alexa-488 experiments, the total time-span of the experiment is approximately 45 seconds, but for the dextran experiments the time-span of the experiment is approximately 130 seconds. Each MW is auto-contrasted in the fields of view to show differences in field of views while having an adjusted LUT for better ease of viewing, with the calibration bar in the top right of each pane. Arrows depict nuclei that are qualitatively heterogeneous to other cells in the field of view, generally for that MW across cell types. (b, e) the population metrics were found as referenced in Figure 1b. The left hand half-violins of each MW correspond to while the right hand half violins of each MW correspond to the normalized fluorescence intensity ($Y_{1/2_{\mathrm{nuclear}}}/Y_{1/2_{\mathrm{peri}-\mathrm{nuclear}}}$). (c, f) Median behavior of the max derivative of the fitted line of each nuclei for all nuclei of a given cell line and MW, where the error bars are standard deviation; note the counts are CellProfiler re-scaled fluorescent counts rather than raw counts from the microscope. Number of cells and experiments are grouped by cell line; MDA-MB-231: alexa-488 *N* = 9 and *n* = 752 cells, 3 kDa *N* = 3 and *n* = 258 cells, 10 kDa *N* = 3 and *n* = 531 cells; PC3: alexa-488 *N* = 5 and *n* = 168 cells, 3 kDa *N* = 8 and *n* = 440 cells, 10 kDa *N* = 7 and *n* = 443 cells. Figure compiled in biorender.

While anecdotal evidence shows that nuclear transport *may* differ across cell lines by a spectrum of LC90s for the same nuclear localizing chemotherapies [27], systematic studies of MW vs nuclear uptake are absent. Many plausible reasons exist for why cells from different tissue may have different nuclear uptake for the same MW cargo. For example, (1) nuclear shape is suggested to impose different diffusion rates [28], (2) different composition or amounts of the nuclear pore complex in different tissues [7–11], or (3) nucleoporin mutations that can asymmetrically affect different MW cargoes, like what has been shown in yeast [3], may occur. Figure 2(a, d) and Figure 3(a, d) depict how individual cell lines show distinct passive nuclear uptake of different MW cargoes.

When looking at the representative fields of view for the different MWs of the individual cell lines in Figures 2(a, d) and 3(a, d), we note that the cytosol appears to be darker than nuclei at the final time point for most MWs and cell lines–meaning the nucleus appears to be a molecular-sink. We would expect the nucleus and cytosol to be equal at equilibrium under *passive diffusion*. The darker cytosol intensity may be due to cytosolic crowding being greater than the nucleus [29], which would drive molecules toward the nucleus, or other reasons (such as the cargo molecules interacting with chromatin or proteins in the nucleus).

Using the previously defined peri-nuclear region as a cytosolic reference–chosen because it has a similar optical thickness to the nucleus within the focal plane–we assess whether cytosolic interactions with the probes occur. As a brief reminder, similar optical thickness is important because we want to compare a similar amount of contribution to ‘out of cell’ cargo fluorescence; conversely, if we used the whole cytosol, that would also incorporate extraordinarily thin sections on the periphery of the cell, which would then incorporate a significantly larger of ‘out of cell’ cargo fluorescence, which would skew our cytosolic response to be immediately equilibrated to the outside fluorescent bath. Median peri-nuclear intensities at the population level $T_{1/2}$ s (Fig. S1) appear consistent across cell lines for specific cargoes. For example, the ratio between the cell line with the highest median peri-nuclear intensity and the lowest for a given cargo is minimal: less than 5 % for both dextrans and around 10 % for Alexa-488. On the contrary, the range of nuclear fluorescence ratios across these cell lines are roughly 4-fold of the peri-nuclear ranges (16–18 %) for the two dextran sizes. The observed increased variations in dextran nuclear fluorescence intensities indicate that nuclear intensity trends are not simply driven by minor fluctuations in peri-nuclear signal. For the Alexa-488 free dye, however, the observation is slightly less straightforward. The Alexa signal had similar max:min ratios for both peri-nuclear and nuclear intensities across cell lines, but the cell line corresponding to one of the two lowest peri-nuclear signals corresponds to the nuclear maximum signal. This indicates that the peri-nuclear intensity is not explicitly dominating the effects seen in the nucleus, as if it were, the nuclear signal for that cell line would have also been the minimum across the cell lines. This consistency across MW that depicts that the peri-nucleus does not dominate nuclear behavior underscores that the nuclear differences reflect more than just noise. Rather, this consistency across MW promotes the idea that the differences in uptake seen across cell lines are nuclear driven.

Across the different cell lines in Figures 2 and 3, there may be multiple aspects that could cause cell-line specific differences in nuclear uptake. For example, number of nuclear pore complexes, cytoplasmic organelle content, nuclear size, nuclear tortuosity, etc may all be different for each cell line. Since these are cell-line-specific features, we further normalize all metrics used across an individual cell line to reduce systematic bias. These ratiometrics are defined as $\mathrm{metri}c_{MW_{\mathrm{High}}}/\mathrm{metri}c_{MW_{\mathrm{Low}}}$ (e.g., any of our metrics such as $Y_{1/2}$ or maximum uptake rate) within an individual cell line. Such a normalization aims to account for these cell-line-specific metrics for each cell line, which enable more robust comparisons of the ratiometric quantities *between* any two cell lines.

Below is an expanded explanation of this ratiometric normalization. The variables are defined prior to the equations:

• N–Number of nuclear pores (constant for a given cell line, but may differ across cell lines, may be any cellular characteristic that is cell-specific)

• $UD_{\mathrm{MW}}$ - Unit diffusion metric for a molecule of a given molecular weight (i.e., the diffusion contribution of a single N to $T_{1/2}$, $Y_{1/2}$, or the maximum nuclear uptake rate; where *N* = nuclear pores for this example)

• $D_{\mathrm{MW}}$ - Total diffusion metric that was measured ($T_{1/2}$, normalized $Y_{1/2}$, or maximum nuclear uptake rate seen in Figures 2 and 3

(2) $$UD_{\mathrm{MW}}*N=D_{\mathrm{MW}}$$

(3) $$\frac{UD_{10\mathrm{kDa}}*100\;\mathrm{nuclear}\;\mathrm{pores}}{UD_{3\mathrm{kDa}}*100\;\mathrm{nuclear}\;\mathrm{pores}}=\frac{UD_{10\mathrm{kDa}}}{UD_{3\mathrm{kDa}}}$$

(4) $$\frac{UD_{10\mathrm{kDa}}*10000\;\mathrm{nuclear}\;\mathrm{pores}}{UD_{3\mathrm{kDa}}*10000\;\mathrm{nuclear}\;\mathrm{pores}}=\frac{UD_{10\mathrm{kDa}}}{UD_{3\mathrm{kDa}}}$$

In this demonstration with Equations 3 and 4, one cell line may have an average of 100 nuclear pores, while the other has 10,000. Given that this is a cell-line-specific characteristic and is independent of molecular cargo, we can isolate the unit diffusion metric through this additional, simple normalization. This allows for the cell lines’ ratiometric uptake metrics to comparable across cell lines. Here, we make a simplifying assumption that the characteristics of ‘N’ will not affect the diffusion of our cargo in a MW-specific way.

**Figure 4.**
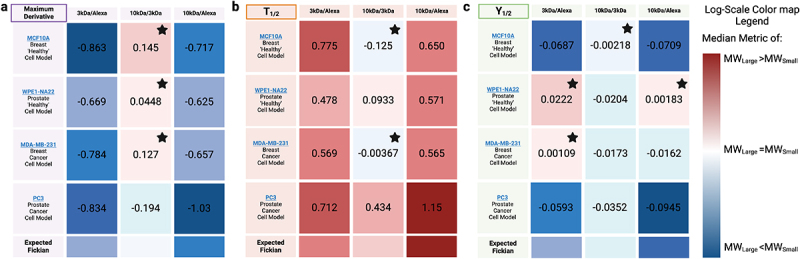
Different cell lines have inherently different uptake proclivities as depicted by cell-line-specific normalization of . Log 10 median values of population metrics of Figures 2 and 3 c, f normalized by different MW per cell line. normalization is the same as in the previous figures, where the nuclear intensity is pre-normalized to peri-nuclear intensity. Red coloring within a box indicates that the numerator variable (the heavier molecular weight) was larger than the denominator variable, where blue is vice versa. Standard passive diffusion color trends are used to compare whether a cell line behaved as one would expect for unhindered passive diffusion across the nuclear membrane. of ‘expected Fickian’ diffusion heatmaps are under the assumption that packing density negatively correlates with molecule size. Heatmap boxes with a black star indicate a non-Fickian response, where the larger molecular weight exhibited a diffusion metric suggesting equal or faster diffusion compared to the smaller molecule. Biological and technical replicates are the same a discussed in the caption of the previous figures. Figure compiled in BioRender.

These MW ratiometric quantities are depicted in Figure 4 as the logarithm of ratios for ease of comparison. If the logarithm is positive (red) the numerator (always the larger molecular weight) is greater than the denominator (always the smaller molecular weight); whereas if it is negative (blue) the denominator is larger than the numerator.

Interestingly, we find that the different cell lines do not all show Fickian diffusion trends for passive transport of MW cargoes up to 10 kDa into the nucleus. Clear examples of atypical diffusion are seen in the ratiometric comparisons of 3 kDa max uptake rate to that for 10 kDa for the MDA-MB-231 (metastatic breast cancer) cell line. Indeed, the same is true for the WPE1-NA22 (a normal prostate epithelial) cell line as their respective 10 kDa max uptake rates were *larger* than the 3 kDa. These results show that passive nuclear entry rate does not monotonically drop with increasing molecular weight, which means passive transport is not purely diffusive–even for relatively small inert dextran cargoes.

Given these results, we performed two controls to verify the non-Fickian behavior was not an artifact of the experiment. (1) An orthogonal permabilization agent, a protein complexing toxin called streptolysin O (SLO) that produces different membrane pores [18–22], was used to verify non-Fickian based-findings see Fig. S2. Indeed, we find that trends between MW and cell lines for the population ratiometrics still show non-Fickian diffusion using the SLOalternative permeabilization for pathological and healthy cells, illustrating the robustness of these metrics. (2) We used giant unilamellar vesicles (GUVs) containing cholesterol as a model membrane mimics [30] through which diffusion can occur if permeabilized to check for aggregation of the MW probes, and verification that the permeabilization technique did not cause aggregation. Diffusion into chemically permeabilized GUVs showed a monotonically increasing $T_{1/2}$ for larger cargoes, as expected (Fig. S3). Using our experimental design, we are capable of resolving differences in diffusion metrics expected, based on increases in MW (for Stoke diffusion) in a simplified system with only permeabilization induced pores in vesicles. Therefore we are confident in our ability to resolve differences in a cargo diffusion into the nucleus in a cellular system if diffusion was the dominant effect. We note that no entrance of any dye is detectable in our GUV experiments without permeabilization.

These controls indicate that neither the use of our permeabilization agents, nor probe molecule aggregation produce the non-Fickian diffusion that we observed within the cells. While GUVs do not explicitly have the cytosolic content seen in cells, we expect the cytosolic content of cells to have been removed through the use of digitonin and multiple washes post digitonin before the dyes are introduced, as previous researchers used similar digitonin concentrations for protocols of cytosolic extraction [26]. Instead, passive nuclear transport of cargoes in this size range appears be affected by additional features that restrict diffusion through the NPC that is size dependent.

### Tissue origin and pathology have a distinct effect on passive diffusion of cargo into the nucleus

When comparing nuclear uptake across molecular weights (MWs) in phenotypically healthy breast and prostate cells, transport differences due to size are less pronounced in prostate WPE1-NA22 cells. Their ratiometric $T_{1/2}$ s are roughly half or less than those of MCF-10A, indicating a smaller size-dependent effect, see Figure 4. This is further supported by the max derivative ratio between 10 kDa and 3 kDa dextrans in WPE1-NA22, showing minimal uptake rate difference, with 10 kDa being slightly faster–an atypical trend if size alone dictated diffusion speed.

A similar tissue-dependent pattern emerges when comparing cancerous cells. In breast tissue, MDA-MB-231 cells show faster 10 kDa uptake relative to Alexa-488 free dye than healthy breast cells, reflected in values closer to zero for both ratiometric max derivative and $T_{1/2}$ (Figure 4). Conversely, prostate tissue shows the opposite trend: healthy cells exhibit faster 10 kDa uptake relative to Alexa-488 free dye than cancerous prostate cells. This reversal across tissue types suggests a pathology-driven alteration in nuclear permeability. Statistical analysis further supports both tissue and pathology dependence: Three-way ANOVAs of our metrics–where tissue, pathology, and MW are the three variables–show significant differences between healthy and cancerous cells, and that pathology and tissue type interact statistically with MW, meaning uptake varies by tissue origin and whether the cell is healthy or cancerous Tables S1 , S2, and S3.

These data show the complexity of transport rates for the various cell lines and MWs. We find no obvious increase in our diffusion metrics for all MW in a given cell line over others. This complexity, coupled with the fact that passive diffusion of cargoes may not follow Fickian diffusion, challenges preconceptions about passive nuclear transport. We note, however, our goal was to investigate if passive nuclear diffusion exhibits variability in different cells rather to create an exhaustive library for all cells and cargo types.

We recognize that up until this point, there are a variety of reasons that could lead to differences in cell uptake of various cargo. In order to limit the number of confounding variables, we used the MCF10A and an induced cell transformation using transforming growth factor-β 1 to form a sort of ‘synthetic matched pair,’ using a well-established protocol [16,24,25,31,32]. This will allow us to better isolate how oncogenesis changes passive nuclear transport since the same cell-type is used for these experiments.

### Oncogenic transformation causes healthy cell nuclear transport to approach cancer cell model

**Figure 5.**
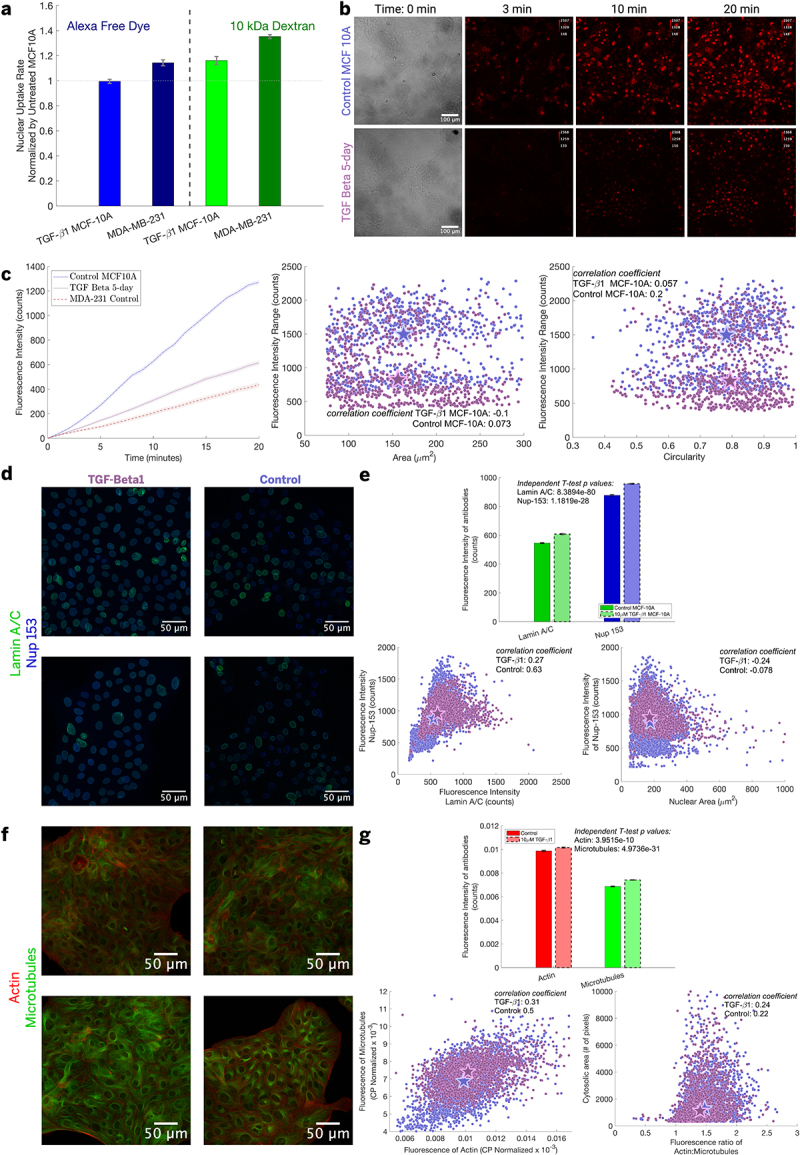
Pathological progression shows a change in nuclear uptake for both inert molecules and a chemotherapy. (a) We use TGF- 1 induced transformation of MCF-10A and MDA-MB-231 to demonstrate a step-wise pathological progression of cell lines. Both uptake rates are normalized to the untreated MCF-10A ‘control’ condition’s average response, all non-TGF- 1 data uses the data from previous figures. Error bars are calculated using propagated standard error between the condition and the untreated ‘control.’ Incubating these different ‘pathological states’ with either Alexa-488 free dye (blue bars) or 10 kDa dextrans (green bars), we can see that the most pathological state (MDA-MB-231) differs from the untreated MCF-10A the most, which is to be expected. However, we also see that the path to transformation progressively appears to change nuclear uptake in a size dependent manner, as the transformed MCF-10A has a different uptake than the untreated MCF-10A for the larger molecular weight. If a bar is along the dotted line at 1 on the y-axis, the ‘pathological state’ of the cell would have no difference to the untreated MCF-10A cell line for that molecule. (b) Representative fields of view of a timelapse of DOX uptake in nuclei for control and TGF- 1 treated MCF-10A–where the fields of view are LUT corrected to show higher contrast, with a calibration bar to show differences between the two treatment conditions. For leftmost subfigure in c, MDA-MB-231 is present in the timelapse quantification for qualitative demonstration purposes that the intermediate transformed MCF-10A is between the untreated and the cancerous MDA-MB-231, but it will not be compared further due to it being an entirely different cell line. The dashed lines are of the median nuclei behavior, and the shading around the median behavior is standard error of that group. The y-axis on all graphs is based on raw fluorescent counts displayed in FIJI. The two graphs on the right of subfigure (c) are depicting that nuclear morphology is not dominantly affecting DOX uptake. (d) Different fields of view depicting heterogeneity of nuclear protein fluorescent antibody labeling of lamin A/C (Alexa-488-green) and an interacting basket nucleoporin–Nup-153 (Alexa-647-blue) for TGF Beta vs control. (e) Quantification of multiple fields of view for the two nuclear proteins showing statistically significant differences between control and TGF-Beta MCF10A, as well as their ratiometric correlation to nuclear area. (f) Different fields of view depicting heterogeneity of cytoskeletal protein fluorescent labeling of actin (phalloidin-594-red) and microtubules (Alexa-488-green). (g) Quantification of multiple fields of view for the two cytoskeletal proteins showing statistically significant differences between control and TGF-Beta MCF10A, as well as their ratiometric correlation to cytosolic area. Color coding for TGF-Beta and control are based on the color their names are displayed with in subfigure (d) and are conserved for all subfigures. Number of cells and experiments are grouped by cell line, for (a) Alexa: MCF-10A control: *N* = 2 and *n* = 210 cells, MCF-10A TGF- 1 5-day: *N* = 2 and *n* = 351 cells 10 kDa: MCF-10A control: *N* = 2 and *n* = 351 cells, MCF-10A TGF- 1 5-day: *N* = 2 and *n* = 302 cells. MDA-MB-231/MCF-10A in (a) use previous figures N and n; for (b-c) doxorubicin: MCF-10A control: *N* = 3 and *n* = 684 cells, MCF-10A TGF- 1 5-day: *N* = 4 and *n* = 709 cells, MDA-MB-231 control: *N* = 5 and *n* = 714 cells; for (e) control: 4 and 3073 cells, TGF: 4 and 2695 cells; for (g) control: 2 and 2160 cells, TGF: 2 and 1881 cells. Figure compiled in biorender.

We tested if cancerous transformation of cells leads to atypical uptake by inducing epithelial-to-mesenchymal transition (EMT) in MCF-10A through a 5-day incubation with transforming growth factor-β 1 (TGF-β 1) [16,24,25,31,32], and then measured nuclear uptake. We used the lowest and highest MW probes in our study as well as the chemotherapeutic doxorubicin (DOX) as a model nuclear-localizing cancer drug. After a 5-day incubation with 10 ng/ml TGF-β 1, cells neared 100% confluence. During incubation with TGF-β 1, we observed minimal changes to morphology, see Figure 5(c). We note that only MCF-10A was used for these experiments as there is no currently accepted protocol for inducing EMT in WPE1-NA22 (as a cell line has to be resistant to the growth suppression aspects of TGF-β 1 like MCF-10A [16,24,25,31,32]).

We found that MCF-10A cells subjected to 5 days of incubation with TGF-β 1 show indistinguishable transport of Alexa 488 (Figure 5(a), blue bars), compared to the control MCF-10A cells. In subfigures a and b of Figure 5, we also include MDA-MB-231 for a *qualitative comparison* to show that the TGF-β 1 cells were an intermediary response between MCF-10A and MDA-MB-231. No quantitative inferences should be drawn from MDA-MB-231 on this graph, as it is an entirely different cell line that was not explicitly normalized by itself, but rather it was normalized by the MCF-10A mean for quick reference.

For instance, the 10kDa dextran nuclear transport in TGF-β 1 cells appears to approach the behavior shown by cancerous MDA-MB-231 Figure 5a, green bars). It is possible that our detection method lacks sufficient sensitivity to distinguish differences between untreated and transformed MCF10A cells for the Alexa-488 molecule, or the possibility that this intermediary cell-transforming cell state does not alter molecule transport at that size. This may be due to the timescale of the diffusion experiment: while the Alexa-488 free dye typically diffuses within 45 seconds, most cell lines reach completion within 10 seconds. In contrast, the 10 kDa dextran has a diffusion timescale of slightly over 2 minutes, with some cell lines requiring the full duration before the dye intensity plateaus.

To test whether small molecule (less than 1 kDa) transport is truly unaffected in this intermediary cell-transforming state, we also evaluated doxorubicin (DOX), a small molecule comparable in size to Alexa 488 but with distinct chemical properties. DOX exhibits significantly brighter fluorescence due to its DNA-binding capability [33,34], and due to its intercalating nature, we found it shows a much slower increase in nuclear fluorescence compared to Alexa 488, which should enhance the sensitivity of our assay. TGF-β 1 treated MCF-10A cells showed considerably reduced DOX uptake, trending toward the MDA-MB-231 behavior (Figure 5(c)). While we do a direct comparison across these two cell lines in this subfigure, it is qualitative and strictly to show that the transformed MCF-10A has an intermediary DOX uptake profile relative to the healthy state of MCF-10A and the cancerous state MDA-MB-231. Subsequent sub-figures *exclude MDA-MB-231*, thereby concluding the inter-line qualitative comparison and shifting focus to the same cell line in either its transformed or parental state. Nevertheless, this result reinforces our hypothesis that cell pathology state influences nuclear uptake of molecules–for both small (∼ 500 Da) and medium (∼ 10 kDa) cargoes. Additionally, in Figure 5(c) *middle* and *right* panels, we note that two populations exist for both cell treatments, similar to our previous work [23]. Additionally, it appears the total fluorescence shifts down for the lower population, which means the already less sensitive population to DOX, will end up having an even lower nuclear uptake rate of DOX with the TGF-β treatment.

Given that the oncogenic transformation of MCF-10A cells altered their uptake to make them more closely resemble the MDA-MB-231 cells, we proceeded to investigate different cytoskeletal and nucleoskeletal proteins that we previously found could alter nuclear uptake [23], as we recognize there are vast differences across the four cell lines analyzed. We found that total cytoskeletal filament expression as well as lamin A/C, and a specific basket nucleoporin that interacts with lamin A/C (purported to influence nuclear pore complex structure and transport [10]), all show statistically significant increases in fluorescent intensity via microscopy (by independent t-tests)–see Figure 5(e-g). The 5-day incubation allows cells to compensate for changes in protein expression–highlighting a similar effect to what we found with culturing cells on soft substrates. Cells have the ability and time to reconfigure their cytoskeletal and nucleoskeletal filamentous proteins, and could change the state of the nuclear pore during this oncogenic transformation in a highly complex manner affecting nuclear uptake in a non-monotonic way. An important thing to note regarding 5e is that the transformed cell line has a 10% greater fluorescent signature; this means that this transformed condition has a higher expression of Nup153, thereby having either more NPCs and/or having altered nuclear pore complex basket conformation–either of which may affect nucleo-cytoplasmic transport [35]. One additional factor that Anton et al. showed [24], that we also expect to contribute in nuclear uptake is cell cycle changes of MCF-10A due to the transformation. Any shift in distribution from G1 to G2 May influence uptake due to nascent nuclear pore formation, in addition to nuclear volume increase. However, we controlled for this by utilizing previous research regarding contact inhibition to impose a cell cycle arrest at G0/G1 with and without TGF-β treatment [36–39]. Given this, the majority of cells should be in a G0/G1 state after 5 days incubation at 100% confluence. Additionally, based on Fig. S7, we would expect a G1 cell to have less uptake than a G2, so changes in nuclear area and cell cycle are not dominating the *decrease* in uptake that the TGF-β treatment induces.

In an effort to put the results of passive nuclear transport from transformed MCF-10A in context, an intermediary cell on the path to oncogenesis could exhibit a 20 % change in molecular transport of ∼ 10 kDa cargo, such as small transcription factors. Additionally, this finding is in line with the general fact that EMT affects larger molecule that undergo facilitated transport [16], albeit for entirely different reasons. As will be discussed in the next section, this also can affect proteins by changing the tendency of nuclear localization due to a change in nuclear permeability (such as mislocalization) [16], which are more commonly seen in cancer cells.

### Small peptide passive transport as a therapeutic drug delivery analog

Both the transformed MCF-10A and MDA-MB-231 that we use as our markers of ‘pathological progression’ showed a sizable change in uptake speed relative to the untreated MCF-10A for larger molecular weights. Building on the observation that oncogenesis can alter passive transport, we attempted to exploit differences seen in **5A** for the larger molecular weight to demonstrate the potential accelerated nuclear uptake of a therapeutic peptide in cancerous cells.

Therefore, we took a small protein-sized fragment of Ap180 (∼ 20 kDa), an adaptor protein in endocytsosis, which has no known interaction partners in cells nor an NLS, as a model peptide to undergo passive nuclear transport [40]. This was an attempt to emulate the weight range of 10–20 kDa proteins are part of a class of therapeutic peptides/proteins that can be used to initiate cell death or control cell transcription [13,14]. For example, molecules such as $p14^{\mathrm{ARF}}$, $p15^{\mathrm{INK}4B}$, and $p16^{\mathrm{INK}4A}$ are tumor suppressors of similar size [41–43]. Thus, if atypical leakiness or restriction exists for transcription factors/tumor suppressors such as these, then cancer cells may be atypically restricted by regulatory factors.

Fig. S8 depicts how this exogenous protein fragment transited across the nuclear membrane of MCF-10A and MDA-MB-231 cells. In Fig. S8b depicting MDA-MB-231 uptake, we have placed arrows to show punctae of the fluorescent peptide aggregation around the nuclear membrane, which caused their normalized metric $Y_{{1/2}_{\mathrm{nuclear}}}/Y_{{1/2}_{\mathrm{peri}-\mathrm{nuclear}}}$ to be skewed. Despite the aggregation in MDA-MB-231 cells, we may still observe that the maximum derivative (**c**) and raw counts (**d**) of Fig. S8 are 5–10% higher for MDA-MB-231 than MCF-10A even for this large protein fragment. Additionally, the metrics discussed are similar to the 10 kDa dextran behavior. This means that this protein fragment is in agreement with the larger dextran, despite being an entirely different structure than the inert sugar molecule.

While we expect for different peptides and proteins of this size to differ in uptake rates and amount, the increased uptake in cancerous cells appears to be consistent even for a protein-like molecule, indicating that the tumor suppressors mentioned above could have different entrance rates for different cell lines, with MDA-MB-231 being *faster* than MCF-10A. This means that one could exploit the increased nuclear uptake to target of nuclear cargoes of this size, similar to the idea of enhanced permeation and retention in tumor vasculature. Intuitively, further studies incorporating *in vivo* work or interactions with tumor microenvironment would be necessary before clinical applications could be realized.

### Limitations and questions for future work

In the following discussion, we acknowledge some of the limitations of our study and look toward paths of future work. First, we focus exclusively on passive nuclear transport. Facilitated transport obviously accounts for a considerable amount of transport across the nuclear envelope, such as for $p14^{\mathrm{ARF}}$ and $p16^{\mathrm{INK}4a}$, which have an NLS [44]. However, for molecules such as these (around 10 kDa), previous researchers took a dextran with and without an NLS and showed import rates into the nucleus were not significantly different for these two probes [45] Therefore, while this study focuses on passive transport, the nuclear localization signal (NLS) may contribute more to the consistency of nuclear import rather than a substantial increase in uptake rate. This interpretation aligns with findings by Naim et al. [45], who demonstrated that multiple passively diffusing molecules can compete with one another for transport into the nucleus. However, if one molecule enters via facilitated transport–such as through an NLS–it does not compete with those undergoing passive diffusion. Thus, the presence of an NLS could help stabilize or buffer transport efficiency, especially in environments crowded with passively diffusing molecules, even if its overall impact on absolute uptake rate is modest. Additionally, we previously noted a phenomenon where cargo was trapped within the nucleus. While this phenomenon is the subject of a follow-up study, this trapping happens only after the cargo has transited into the nucleus.

We also attempt to use our statistical findings of pathology impacting nuclear uptake by validating it with the MCF10A transformation previously used by other researchers [16,24,25,31,32]. We leverage previous extensive characterization of EMT induction on MCF-10A with TGF-β, which highlights the accepted and established nature of this protocol. Between the two cell lines used as healthy cell models, only MCF-10A is well categorized for EMT, and so prostate cells were not considered in the oncogenic transformation or subsequent studies. Using this transformation, we can limit the larger differences from across cell lines in an effort to glean some understanding on how pathological transformation may affect nuclear uptake. While we compare treated (seen as an intermediate pathological progression) and untreated conditions (seen as the healthy condition), we also compare this to MDA-MB-231 which is an entirely different–fully invasive and cancerous–cell line. This was done to give us the extreme condition of a fully transformed condition, to show whether the intermediary transformation showed a nuclear uptake trend towards or away from a cancer cell line. While we acknowledge the challenges of direct comparison, we find the pathologic:healthy ratiometric comparison useful to demonstrate the finding that oncogenesis affects passive nuclear uptake. Moreover, we recognize that statistical significance does not inherently imply biological relevance, and our aim here is to demonstrate that uptake rates vary significantly across cell lines and pathological states. In some cases, the uptake rate of a given molecule is reduced by half–or more–compared to other cell lines. Although this observation does not directly confirm a functional consequence, it provides important insight into potential uptake features that may be explored as a ‘sieve effect’ in future studies. At the core of our findings is the proposition that a faster rate of nuclear entry for a molecule reduces its exposure time in the cytoplasm. Therefore, our study highlights (through the use of doxorubicin and other exogenous small molecules) the likelihood of successfully nuclear transit, represents the reciprocal outcome to drug efflux pump–mediated removal. This finding is exaggerated across tissue origins of our study, which lends tentative support to previous claims stating nucleoporins have a key role in cell differentiation [15], as cell differentiation is regulated by transcription factors requiring entrance to the nucleus.

In explaining the differences between transformed MCF-10A and normal MCF-10A cells uptake of DOX or 10 kDa dextrans, we found lamin A/C, a known cancer biomarker, expression changes and cytoskeletal expression/reorganization, occur during the epithelial to mesenchymal transition [46,47]. Additionally, lamin is known to be expressed uniquely in different tissues, which may be a contributor to the tissue dependence on uptake. However, by no means are these the only relevant differences to explain this complex transport question. One facet that is relevant to both the transformed cell line and different tissue types is cell cycle. We have conducted preliminary studies showing that NPC permeability is affected by cell cycle phase, where passive transport increases in G2 compared to G1. As we emphasize here, different cell lines may differ in the distribution and duration of cell cycle stages due to genetic or epigenetic alterations. EMT induction from healthy epithelial cells can occur, as such cell cycle distribution was shown to happen by transformation of MCF-10A cells [24]. Mechanistically, we believe cell cycle [24], the mechanical stressors and cell compensation [23] are some key players of nuclear uptake based on current and previous work, but are not the only contributors.

Because TGF-β is known to affect full cytoskeletal structure and content [46,47], we ran an experiment to determine if a correlation exists between nuclear uptake of DOX and actin content using MDA-MB-231 cells, see **Fig. S10**. We found a weak correlation, $R^{2}$ = 0.37 between actin fluorescence and nuclear uptake of DOX across the same cells in a field of view. These results agree with our previous work showing that non-acute mechanically reinforced cells exhibit larger DOX uptake. In the current study where a 5-day TGF-β treatment was used to induce oncogenesis in MCF-10A cells, we observed that cytoskeletal staining of actin and microtubules increases by less than 10 % while DOX nuclear uptake decreased. This further exemplifies that nuclear permeability is affected by many processes, and that cellular changes due to oncogenic signaling may overwhelm changes in the cytoskeletal structure and content.

With this information and our TGF-β finding, our hope is to spark a larger scientific interest in the exploration of nuclear permeability as a complex variable within both biological questions and drug delivery in future works.

Additionally, we do not expect all invasive or malignant nuclear uptake would be the same for all cell types in a tumor or even sub-types of cancer, due to the same factors that we put forth that contribute to nuclear uptake. For example, even though MDA-MB-231 was used as a model for the fully invasive cancer condition, it is not reasonable to assume that it would be an exact intermediate response for any and all invasive malignant breast cancer subtypes, based on the mechanistic contributing factors that current and previous research has highlighted.

We investigated other facets of the cells, specifically nuclear morphology (area and circularity) and cell morphology (cobblestone or spindle shaped) and found that none appeared to correlate with a change in nuclear uptake of DOX (see Figure 5e-g and Figure S6), which contrasts with computer modeling of previous research [28].

If the 20 kDa peptide and 10 kDa dextran are representative of a range of small proteins, such as the tumor suppressors discussed earlier, the increased nuclear uptake of tumor-suppressing proteins raises an interesting question to be explored in the future: *Why aren’t cancer cells that exhibit higher nuclear uptake of these small tumor suppressor proteins, more significantly affected by them?* While this goes beyond the scope of this study, this may incidentally reflect the evolutionary pressures that cells like MDA-MB-231 must overcome. Previous research notes that mutations/overexpression of $p14^{\mathrm{ARF}}$ and $p16^{\mathrm{INK}4A}$ [43,44] proteins exist in cancer cell lines as well as various types of tumors, which result in a functional change in the proteins, where they appear to behave in a manner opposite to that of the normal proteins’ role.

We hope that analyzing specific MW uptake may reveal a MW ‘window’ that can be used to aid in the design of future size-specific drug delivery studies for specific cancer types, similar to the enhanced permeation and retention effect for leaky vasculature. For instance, numerous experiments highlighted the preferential entrance of 10–20 kDa cargoes into the nucleus of breast cancer cells compared to normal cells. Additionally, Figure 5 indicates that the rate of entrance and normalized fluorescence intensity of 10 kDa is larger for MDA-MB-231 relative to its respective healthy cell line, and may be a more advantageous size than smaller molecules such as Doxorubicin, which showed less uptake in MDA-MB-231 compared to MCF-10A. However, we also learned that passive nuclear uptake appears to be tissue-type specific. Because of the difference in breast vs prostate cells, our data tentatively suggests that the same molecular therapeutic would not be used in both tissues. In prostate tissue, where normal cells exhibit more nuclear uptake than cancerous cells at 10 kDa, such a larger molecule would be a poor target choice while it may do well in breast cancer. However, being able to predict susceptibility to a given molecular weight, and the fact that it may have tissue specificity for advantageous delivery could help guide new therapeutic development.

We recognize the need for rigorous in vivo studies, and studies focused on tumor microenvironment effects on nuclear uptake must be done to show clinical relevance of our findings. However, limitations of the digitonin protocol to bypass the plasma membrane currently only allow for in vitro studies. Future technologies allowing such work in vivo can help alleviate this need to bridge the gap between this basic science and drug delivery finding to clinical sciences.

## Conclusion

Nuclear transport is an important regulatory process for cell phenotypic behavior, and we have shown here that transport differs in both pathology and tissue type for various molecular weights. Passive transport has previously been correlated with Brownian motion, but standard diffusion laws did not appear to hold true across all cell lines, as larger molecular weights (10 kDa) seemed to be just as likely to cross the nuclear membrane as smaller ones (700 Da) for some cell lines.

We demonstrate that evaluating multiple cell lines for a given nuclear transport phenomenon is prudent, as trends observed in a single cell line are not consistently conserved across different tissue types and pathological conditions. By establishing this precedent, we aim to enhance the rigor of future nuclear trafficking studies, given that individual cell lines are often analyzed in isolation. Additionally, we found that inducing EMT in a normal-like breast epithelial cell line pushed the passive nuclear uptake characteristics toward the response of that observed for invasive, mesenchymal cells. Therefore, we believe passive nuclear transport is modulated in the process of oncogenesis.

To validate our results showing preferential uptake of 10 kDa dextrans in breast tissue cells, we used a protein fragment from Ap180 that was larger than 10 kDa to depict a range of molecular weights of potential beneficial therapeutics and saw a faster uptake for the cancerous cells compared to healthy model. While this consistency is exciting, it begs a future question about how small molecular weight proteins that are tumor suppressors appear to be less effective in specific cancerous cell lines.

## Supplementary Material

Supplemental Material

## Data Availability

All data used to generate the figures in this manuscript are available in an open-access Data Repository: https://doi.org/10.17617/3.N1QH9K

## Abbreviations

Dox: – Doxorubicin HCl,
NPC: - Nuclear Pore Complex,
NLS -: Nuclear Localization Sequence,
EMT: - Epithelial-to-Mesenchymal Transition,
MW: - Molecular Weight,
SLO -: Streptolysin O,
kDa: - kilodaltons,
Nup: – Nucleoporin,
RD2 -: RedDot2,
GUV -: Giant Unilamellar Vesicle
