# Supplementary material for: Passive nuclear transport deviates from Fickian behavior in prostate and breast cell types
Source: Nucleus. 2026 Jan 31;17(1):2620223. doi: 10.1080/19491034.2026.2620223 (PMC12867411; doi:10.1080/19491034.2026.2620223)
Supplement: Supplemental Material [file KNCL_A_2620223_SM4699.zip › Alt text for figures.docx]

Alt text for figures

F1: Experimental overview demonstrating how molecule uptake was quantified for nuclear uptake in cells. A schematic of the experiment, and a demonstration showing raw data and the metrics pulled from it, as well as an example of a field of view over time showing fluorescent uptake is present.

F2: Various molecular cargo is shown over time for the same fields of view to demonstrate the ability for the specific cargo to enter the nucleus of a cell for two different cell lines. Subsequent subfigures are the quantification of the fields of view shown, to demonstrate that each cargo has a unique entrance to the nucleus for the two healthy cell lines.

F3: Various molecular cargo is shown over time for the same fields of view to demonstrate the ability for the specific cargo to enter the nucleus of a cell for two different cell lines. Subsequent subfigures are the quantification of the fields of view shown, to demonstrate that each cargo has a unique entrance to the nucleus for the two cancer cell lines.

F4: A heatmap that is ratiometrically normalized depicts the unique aspects of cargo entrance across four different cell lines across 3 different cargo sizes. It demonstrates that cargo is not always explicitly fickian diffusion controlled, and each cell line has a unique nuclear permeability across cargo sizes.

F5: A demonstration of transforming a cell line and comparing it to the non-transformed version of itself yields different nuclear permeability for different cargo sizes. Immunofluorescence was then used to determine whether different protein components of the cell may have changed during this transformation to help explain why differences exist.

*Supplemental document:*

SF1: Graphs depicting the fluorescent cargo in the nucleus relative to surrounding the nucleus are used to demonstrate that nuclear intensity of cargo is not explicitly dominated by surrounding nuclei intensity. This is shown for all sized cargos and is a consistent feature.

SF2: As a control for digitonin permeabilization, a second permeabilization technique was used to verify findings. These graphs depict that for the two different permeabilization techniques, similar trends across cargo for two cell lines for all sized cargos were consistent.

SF3: As a control for membrane permeabilization and non-fickian behavior, simplified vesicles were used with permeabilization. This figure demonstrates the entrance of dye into these simple vesicles, and that the rates across this simplified system were as expected—fickian dominated.

SF4: Brightfield images of the healthy cells to demonstrate morphological differences across the two healthy cell lines.

SF5: Brightfield images of the cancer cells to demonstrate morphological differences across the two cancer cell lines.

SF6: A control for morphological disturbance with any treatment used in this paper. This figure depicts two different fields of view that show untreated and vastly different morphological changes due to treatment using a different drug. Subsequently, this also shows how there is minimal uptake change for the two different conditions.

SF7: A control that factors in cell cycle phase. An uptake study was done when factoring in whether cells were in cell phase G1 or G2, and the bar graphs depict that G2 consistently had greater permeability. The field of view shown is to depict how one determines whether a cell is in G1 or G2, based on the fluorescent signature it is emitting.

SF8: A demonstration using a small peptide fragment that shows the two cell lines have different permeabilities to larger peptide sized molecules. Fields of view over time are shown depicting the molecules entrance into the nucleus as well as a quantification of the metrics for the two cell lines.

SF9: Zoomed in images depicting two proteins, Lamin A/C and Nup153, for the transformed and untreated cell line. This is to demonstrate there are no drastic changes to protein folding or distribution in the two cell treatment conditions.

SF10: A control using actin immunofluorescence was paired with a live cell nuclear uptake study to show a correlation between the two different parameters. A weak positive correlation exists.
