# Supplementary material for: Passive nuclear transport deviates from Fickian behavior in prostate and breast cell types
Source: Nucleus. 2026 Jan 31;17(1):2620223. doi: 10.1080/19491034.2026.2620223 (PMC12867411; doi:10.1080/19491034.2026.2620223)
Supplement: Supplemental Material [file KNCL_A_2620223_SM4699.zip › SI_of_NucCellSpecific.pdf]

Passive Nuclear Transport Deviates from Fickian Behavior in Prostate and Breast Cell Types: supplemental document

Naming Convention for Countable Items

Figure S1
Figure S2
Figure S3
Figure S4
Figure S5
Figure S6
Figure S7
Figure S8
Figure S9
Figure S10
Table S1
Table S2
Table S3
Table S4
Table S5
Table S6

FIGURES

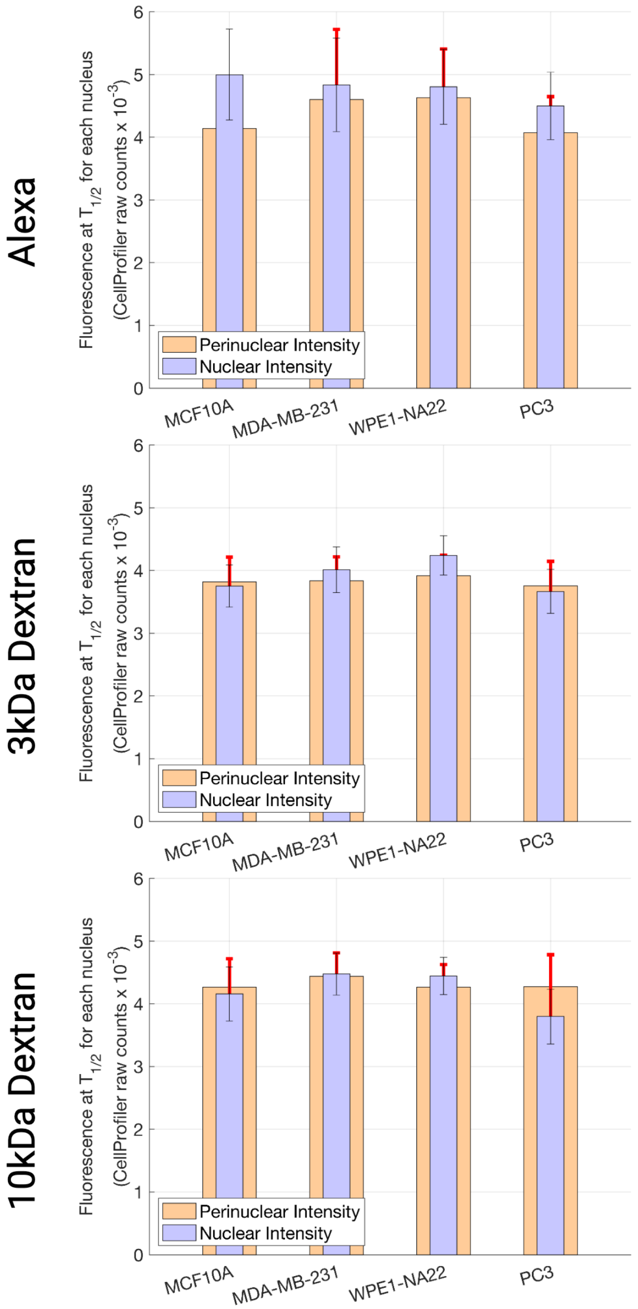

Figure S1. Raw peri-nuclear signal compared to nuclear signal for all cell lines and MWs show that the cytosol does not restrict the dye abnormally. Instead, differences highlighted in the main text are reinforced by the discrepancies of nuclear vs perinuclear differences for each cell line. Note that some deviation on perinuclear is to be expected, as different cell heights and cell shapes may lead to differing exclusion of the 'extracellular' excess dye. The y-axis on this graph is based on CellProfiler re-scaled fluorescent counts.

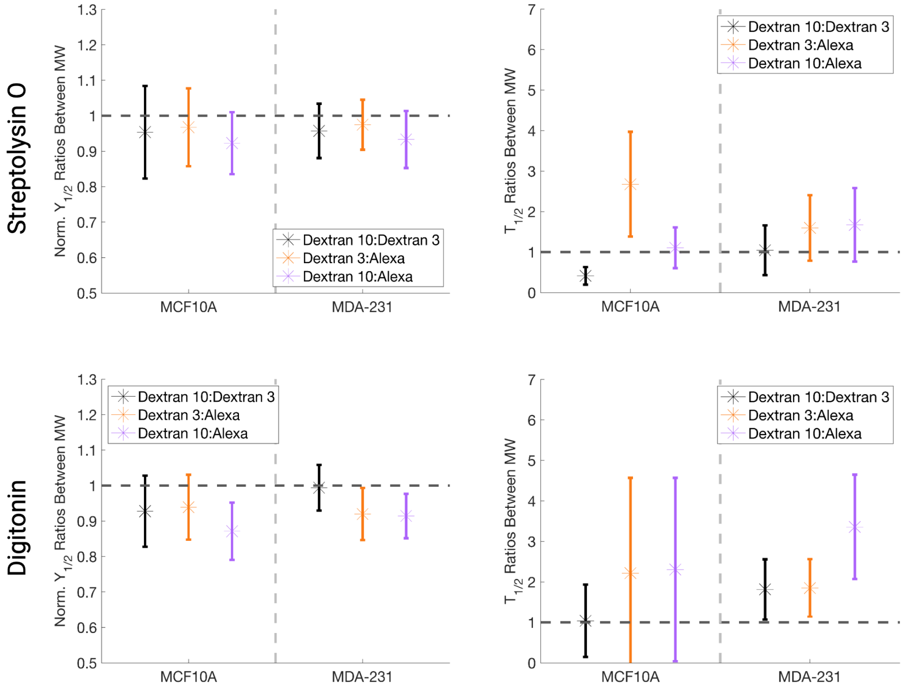

Figure S2. Permeabilization method is does not affect trends seen across MW behaviors in both healthy and cancerous breast tissue. While permeabilization method SLO creates larger pores than digitonin which may affect rate, ratiometric normalization and comparisons between methods are shockingly consistent. This also indicates that the phenomenon measured and ratiometrically compared is not explicitly biased by our permeabilization, but is in an cell characteristic unique for each MW. Note that this data is not what is referenced in the heatmaps, because that is log based ratios vs raw data ratios displayed here.

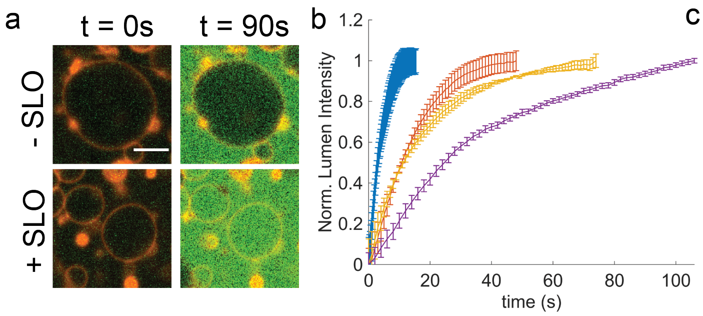

c

Size	$t_{1/2}$
Monomer	3.3
3 kDa	11.3
10 kDa	13.9
40 kDa	28.1

Figure S3. SLO permeabilization study in DOPC Vesicles shows normal Fickian diffusion laws into a lipid membrane compartment. (**a**) Representative images of Alexa488 conjugated 10kDa dextran uptake in vesicles. Scale bar = 5 µm. (**b**) Time course studies of dye uptake within vesicles of Alexa488 (blue), Alexa488-3kDa Dextran (Orange), Alexa488-10kDa Dextran (Yellow), and Alexa488-40kDa Dextran (Purple). Vesicle luminal values were normalized to the outer solution. Error bars represent averages of averages using 3 vesicles (n=3) from triplicate experiments (N=3). (**c**) A table of $T_{1/2}$ for each MW. Similar $T_{1/2}$ analysis was conducted by polynomial fitting to the curves from B.

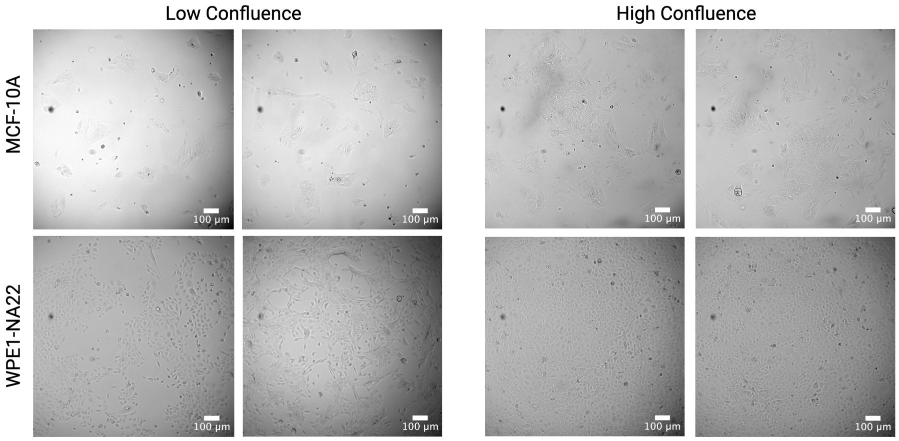

Figure S4. Bright field images under different confluences to show consistent morphological phenotypes these healthy cells exhibit. Cobblestone-like phenotypes are prominent in both low and high confluence for both cell lines.

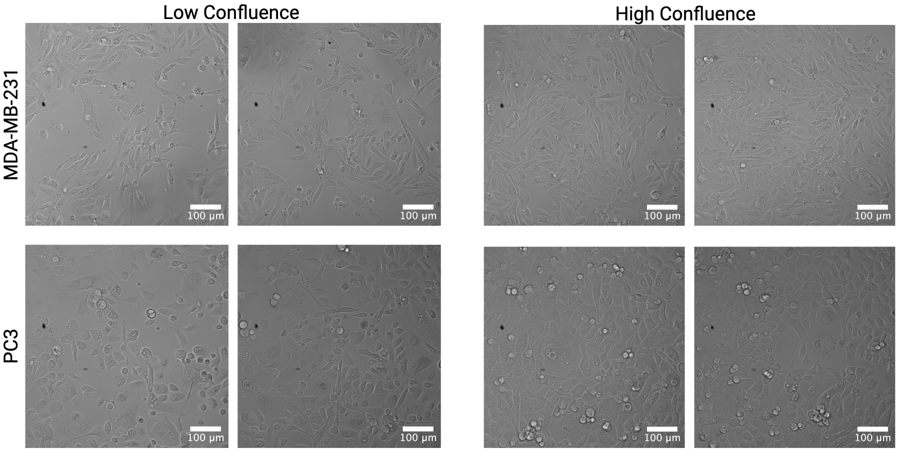

Figure S5. Bright field images under different confluences to show different morphological phenotypes these cancerous cells exhibit. MDA-MB-231 cells show spindle-shaped cells regardless of confluence, whereas PC3 has spindle and cobblestone-like cells, weighing more heavily on the latter as confluence increases.

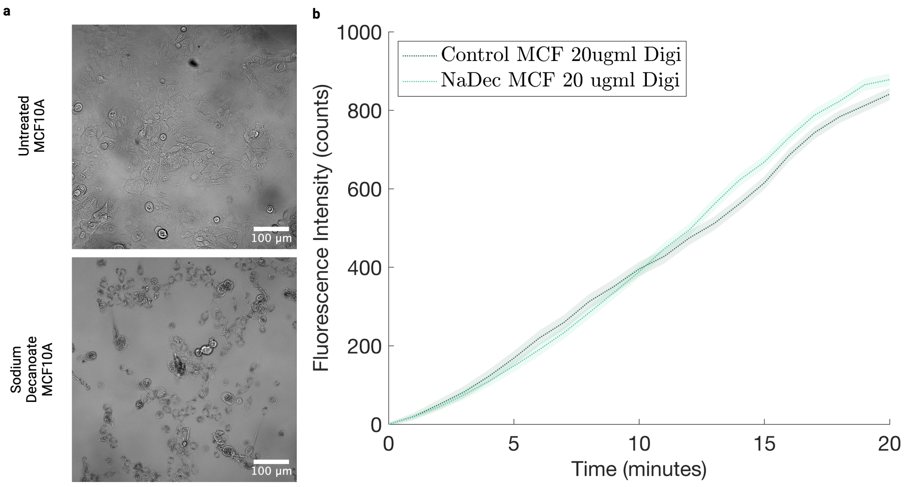

Figure S6. Morphology perturbation does not necessarily cause sensitization of nuclei to DOX. (**a**) Bright field images under different treatment conditions to show different morphological phenotypes that MCF10A exhibits when Zonula Occluden protein, which are tight junction proteins, are dilated using sodium decanoate. Treated cells look much more elongated, more closely resembling MDA-MB-231 cells than the control cells do. (**b**) Time-lapse comparison of the median behavior (line) and standard error (shading) of sodium decanoate. The y-axis is based on raw fluorescent counts found in FIJI. N and n of untreated MCF10A are 2 and 407 cells, while sodium decanoate treated cells are 2 and 472 cells.

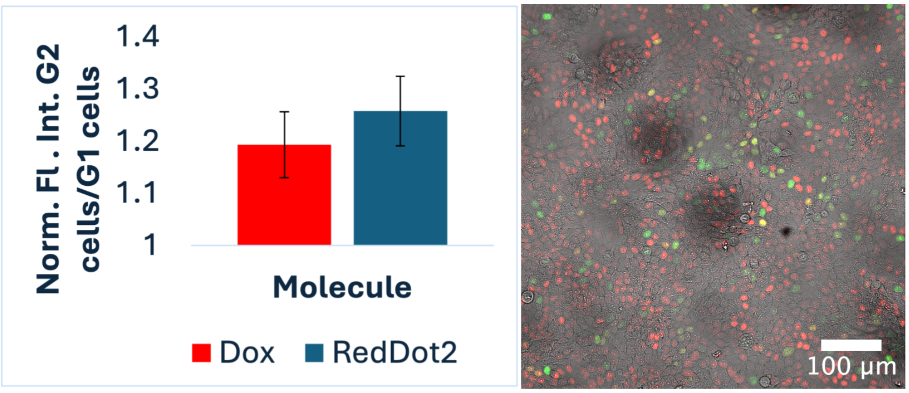

Figure S7. Average nuclear fluorescence of intercalating fluorescent cargoes for cells in G2/G1. (left) HCC-1806 cell line stably transfected with a fluorescent reporter protein (FUCCI) to indicate if cells are in G1 or G2+ of the cell cycle shows increased uptake within G2 cells compared to G1 for two cargoes. Both doxorubicin and RedDot2 fluorescent dyes display more uptake for cells in G2. N =4 for both Dox and RD2. Error bars are standard error, calculated based on the averages of each replicate experiment. (right) Cells in both G1 and G2 are seen in every field of view, and ratios are quantified for each experiment respectively. Note the colors shown are from the cell phase reporter. Green is a fluorescent protein (geminin) prominent within the nucleus during G2, while Red is a fluorescent protein (CDT 1) prominent within the nucleus during G1.

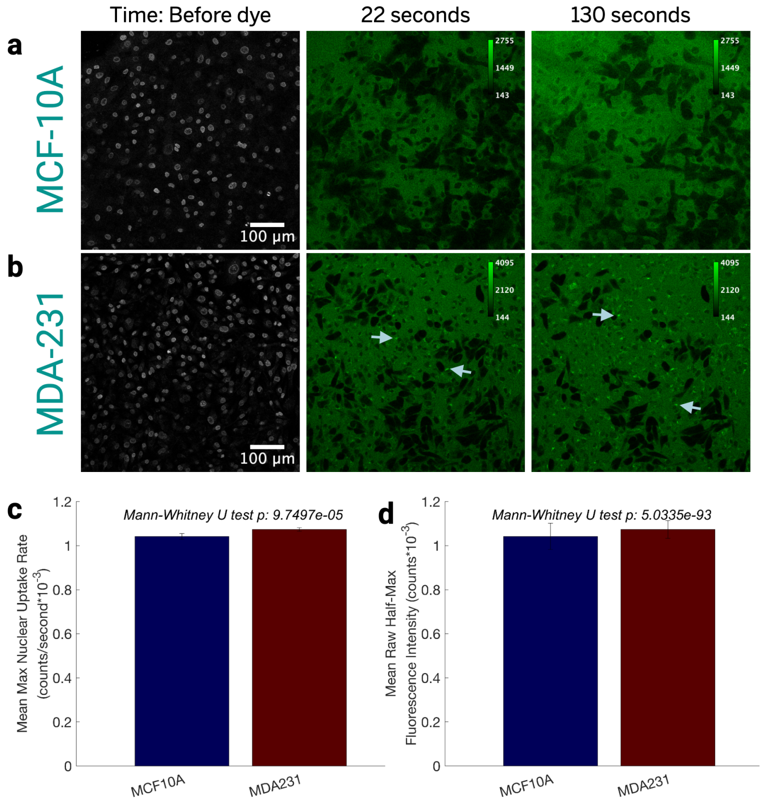

Figure S8. An exogenous Atto-565 labeled peptide approximately at 20 kDa shows different uptake in cancer and healthy cells of the breast cell lines. (**a-b**) Snapshots at different time-points showing uptake into nuclei for MCF-10A and MDA-MB-231 cell lines of this exogenous peptide. Arrows around the nuclei in the MDA-MB-231 cell line show punctae of this peptide accumulating near the nuclear membrane, which skews nuclear vs cytosolic ratios. Both time lapse fields of view were auto-contrasted to show differences in field of views while having an adjusted LUT for better ease of viewing, with the calibration bar in the top right of each pane. (**c-d**) Bar charts of the mean max nuclear uptake and mean raw uptake of the two cell lines, both are statistically different using the Mann-Whitney U test. Error bars are standard error. Number of cells and experiments are grouped by cell line; MCF-10A: N=7 and n=1,374 cells, MDA-MB-231: N=13 and n=3,056 cells. Figure compiled in biorender.

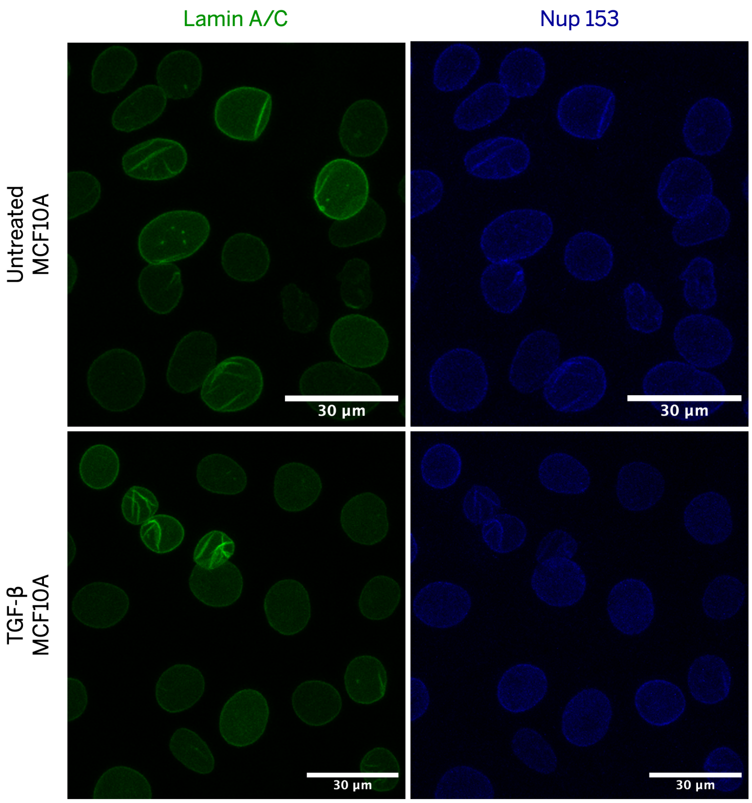

Figure S9. Optical zooms of images of untreated MCF10A (top) and TGF-β-treated MCF10A (bottom) for lamin A/C (in green) and Nup153 (in blue). Note nuclei in these zoomed fields of view show a small percentage of nuclei with folds, but across all images, the percentage of folding is consistently low (less than 5%) for both cases. Figure compiled in biorender.

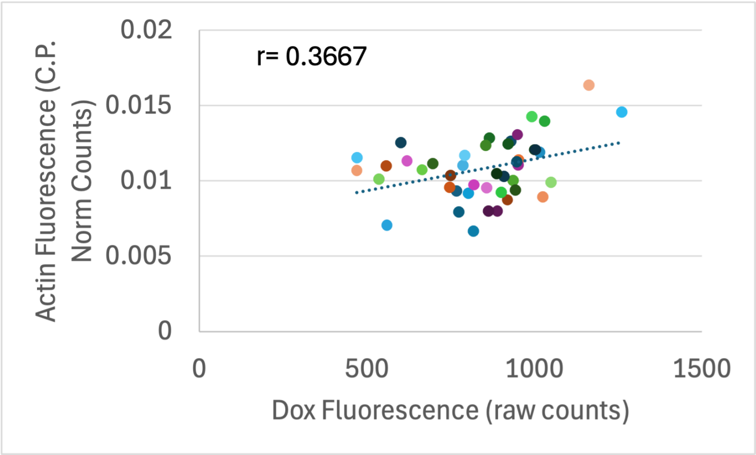

Figure S10. Analysis of MDA-MB-231 single cell DOX internalization over 20 minutes and subsequent actin staining. A weak positive relationship appears to exist between the two F.I. measurements. Protocols used were consistent with those protocols in the main text, aside from digitonin concentration, as we wanted to use every cell in the field of view as a data point for this correlation study. n=43 cells.

TABLES

Table S1. 3-Way ANOVA of $T_{1/2}$ and Interactions (notated as :) between Tissue, Pathology and Molecular Weights

Source	Mean Square	Probability
Pathology	1243	0
Tissue	2.1	0.7857
Molecular Weights	12519.6	0
Pathology:Tissue	30994.3	0
Pathology:MW	1080.2	0
Tissue:MW	6.1	0.8067

Table S2. 3-Way ANOVA of $Y_{1/2}$ and Interactions (notated as :) between Tissue, Pathology and Molecular Weights

Source	Mean Square	Probability
Pathology	0.81507	1.595e-23
Tissue	0.20663	4.451e-7
Molecular Weights	3.717	2.693e-186
Pathology:Tissue	6.110	1.214e-156
Pathology:MW	0.565	1.053e-30
Tissue:MW	1.235	2.315e-65

Table S3. 3-Way ANOVA of Max Derivative and Interactions (notated as :) between Tissue, Pathology and Molecular Weights

Source	Mean Square	Probability
Pathology	0	0.153
Tissue	0	0
Molecular Weights	0.00183	0
Pathology:Tissue	0.00008	0
Pathology:MW	0	0.0537
Tissue:MW	0	0

Table S4. **2-Way ANOVA of $T_{1/2}$ and interactions of Cell Lines and Molecular Weights**

Source	Mean Square	Probability
Cell Line	12582.3	2.56e-307
Molecular Weights	12212.9	3.01e-208
Interaction	5309.6	4.13e-260

Table S5. **2-Way ANOVA of $Y_{1/2}$ and interactions of Cell Lines and Molecular Weights**

Source	Mean Square	Probability
Cell Line	2.544	0
Molecular Weights	3.883	0
Interaction	2.876	0

Table S6. **2-Way ANOVA of Max Derivative and interactions of Cell Lines and Molecular Weights**

Source	Mean Square	Probability
Cell Line	0.0002	0
Molecular Weights	0.00185	0
Interaction	0	0
